# Editorial: The role of gene mutations in the neuropathology of amyotrophic lateral sclerosis (ALS) and frontotemporal lobar degeneration (FTLD)– Progress and challenges

**DOI:** 10.3389/fneur.2023.1145823

**Published:** 2023-02-28

**Authors:** Claire Troakes, Francesca Luisa Conforti

**Affiliations:** ^1^Department of Basic and Clinical Neuroscience, Institute of Psychiatry, Psychology and Neuroscience, King's College London, London, United Kingdom; ^2^Department of Pharmacy, Health and Nutritional Sciences, University of Calabria, Rende, Italy

**Keywords:** amyotrophic lateral sclerosis (ALS), frontotemporal lobar degeneration (FTLD), neuropathology, gene mutation, familial

Over the last decade, there has been an increase in research surrounding the specific pathologies related to gene mutations in ALS and FTLD. It has long been recognized that familial forms of these diseases can show hallmark pathologies related to mutations, e.g., ALS-FUS or FTLD-MAPT. This has been expanded recently by the identification of the C9orf72 expansion and its characteristic pathology (1). However, much remains to be understood regarding the relationship between gene mutations, disease pathology, and clinical manifestation. Some gene mutations result in aggregates of the protein product whereas others appear to cause a loss of function that alters downstream pathways and affects processes such as RNA biology. Thorough neuropathological assessment of common and rare or newly discovered gene mutations may lead to a further understanding of the disease processes in familial and sporadic diseases. Insights into gene-linked pathology may open new therapeutic targets for these currently untreatable diseases. An interesting example is provided by two recent works that describe three key proteins involved in the disease development of ALS and frontotemporal dementia caused by the C9orf72 mutation that can be targeted by drugs (2, 3). Furthermore, the flood of genetic information provided by exome and whole genome sequencing studies over the last 10 years, while useful, has also made distinguishing between truly disease-causing mutations and rare, non-pathogenic polymorphisms in newly identified genes (and indeed in known genes) an inherent challenge. As the majority of inherited and sporadic ALS cases possess a common downstream TDP-43 pathology, confidently determining true upstream genetic causes is critical. Clinicians are calling for more genetic testing in apparently sporadic ALS cases (4) and this, alongside continued expert neuropathological assessment, will expand the understanding of the pathological mechanisms of these diseases.

We have therefore collected, in this Research Topic, a number of papers that cover various aspects of neuropathology, pathophysiology, biomarkers, and genetics related to ALS and FTD. Henderson et al. discuss the evolution of the frontal lobes, metabolic adaptations during human evolution, and the consequences of aging on neuronal processes, linking them to known ALS/FTD pathomechanisms, mainly those related to RNA/protein turnover. The authors propose that neurodegenerative processes affecting the frontal lobes, specifically ALS and FTD, may result from a mismatch between cellular and metabolic pathways and increasing lifespan within an evolutionary framework.

A general overview of biological networks and complexity in early-onset Motor Neuron Diseases (MNDs) is given by Butchbach and Scott. They offer their perspective on novel conceptual frameworks which could help find common therapies for MNDs. They point out how research has so far mainly focused on investigating the pathomechanisms linking mutations in a gene to a certain MND and propose an alternative system biology approach to understand early-onset MNDs, paving the way for new therapeutic possibilities for these diseases.

This Research Topic also concentrates on the role of causative genes shared in ALS and FTD, highlighting possible therapeutic approaches for these gene mutation-linked diseases. Scarian et al. report recent findings on the role of the valosin-containing protein (VCP) gene, an ATPase involved in many different biological functions including protein degradation, autophagy, and lysosomal and mitochondrial homeostasis. The authors report evidence of possible therapeutic approaches targeting the VCP pathway, describing the use of many different drugs already developed for the treatment of other diseases, such as VCP inhibitors able to: (i) prevent muscle cell death and restore muscle integrity and mitochondrial size in VCP mutants; (ii) increase the oxygen consumption rate of patient's fibroblasts; and (iii) reverse the mislocalization of TDP-43 and FUS in mutant motor neurons.

Additionally, the field shows interest in studying the role of neurofilament light chain (NfL) as a biomarker in ALS that can provide a deeper understanding of the pathophysiological mechanisms and potentially effective therapies for this disease. In their prospective cross-sectional study, Zhang et al. investigate the correlation between serum NfL levels and the severity of lower motor neuron (LMN) axonal degeneration in patients with ALS, particularly in the early stages of the disease. The authors show that the use of NfL blood levels reflects the extent of limb LMN axonal damage but not upper motor neuron involvement among ALS patients, indicating that the use of this biomarker may have a profound effect on patient selection and efficacy monitoring of treatment in disease-modifying clinical trials.

The four articles included in this Research Topic provide an overview of the current state-of-the-art research on the role of gene mutations in the neuropathology of ALS and FTD, ranging from characterizing the pathomechanisms related to a specific genetic variation to re-evaluating biomarkers useful for the stratification of patients and directing the search for disease-modifying therapies. Much has been done and much needs to be done to better describe the gene-linked pathology in ALS and FTD. Traditional and new technologies still show many limitations, for example, in detecting the correct size of large expansions of C9ORF72, and better biomarkers are needed. The next decade promises to shed light on new aspects of the relationship between gene mutations, disease pathology, and clinical manifestation in ALS and FTD, enabling improvements in treatment and the finding of effective cures for these devastating diseases.

## Author contributions

Both authors listed have made a substantial, direct, and intellectual contribution to the work and approved it for publication.
